# Inhibition of 26S Protease Regulatory Subunit 7 (MSS1) Suppresses Neuroinflammation

**DOI:** 10.1371/journal.pone.0036142

**Published:** 2012-05-18

**Authors:** Wei Bi, Xiuna Jing, Lihong Zhu, Yanran Liang, Jun Liu, Lianhong Yang, Songhua Xiao, Anding Xu, Qiaoyun Shi, Enxiang Tao

**Affiliations:** 1 Department of Neurology, Sun Yat-sen Memorial Hospital of Sun Yat-sen University, Guangzhou, People’s Republic of China; 2 Department of Neurology, First Affiliated Hospital of Jinan University, Guangzhou, People’s Republic of China; 3 Joint Laboratory for Brain Function and Health of Jinan University and the University of Hong Kong, School of Medicine, Jinan University, Guangzhou, People’s Republic of China; 4 Division of Cardiovascular Medicine, Center for Inherited Cardiovascular Disease, Stanford University School of Medicine, Stanford, California, United States of America; University of North Dakota, United States of America

## Abstract

Recently, researchers have focused on immunosuppression induced by rifampicin. Our previous investigation found that rifampicin was neuroprotective by inhibiting the production of pro-inflammatory mediators, thereby suppressing microglial activation. In this study, using 2-dimensional gel electrophoresis (2-DE) and mass spectrometry (MS), we discovered that 26S protease regulatory subunit 7 (MSS1) was decreased in rifampicin-treated microglia. Western blot analysis verified the downregulation of MSS1 expression by rifampicin. As it is indicated that the modulation of the ubiquitin-26S proteasome system (UPS) with proteasome inhibitors is efficacious for the treatment of neuro-inflammatory disorders, we next hypothesized that silencing MSS1 gene expression might inhibit microglial inflammation. Using RNA interference (RNAi), we showed significant reduction of IkBα degradation and NF-kB activation. The production of lipopolysaccharides-induced pro-inflammatory mediators such as inducible nitric oxide synthase (iNOS), nitric oxide, cyclooxygenase-2, and prostaglandin E_2_ were also reduced by MSS1 gene knockdown. Taken together, our findings suggested that rifampicin inhibited microglial inflammation by suppressing MSS1 protein production. Silencing MSS1 gene expression decreased neuroinflammation. We concluded that MSS1 inhibition, in addition to anti-inflammatory rifampicin, might represent a novel mechanism for the treatment of neuroinflammatory disorders.

## Introduction

Microglial activation plays an important role in the pathophysiology of neurodegenerative diseases, and the suppression of microglial activation has been shown to prevent the progression of Alzheimer’s disease (AD), Parkinson’s disease (PD), trauma, multiple sclerosis, and cerebral ischemia [1]–[5].

Rifampicin is a macrocyclic antibiotic that is used extensively against Mycobacterium Tuberculosis and other mycobacterial infections [6]. It has been reported that rifampicin is immunosuppressive [7]–[10]. We previously found that rifampicin improved survival of catecholamine and α-synuclein-containing cells, which degenerate in PD, thus might be therapeutic in this disease [11]. Rifampicin suppressed the release of pro-inflammatory mediators including nitric oxide (NO), prostaglandin E_2_ (PGE_2_), tumor necrosis factor-α (TNF-α), and interleukin-1β (IL-1β) from BV2 microglial cells that were pre-treated with lipopolysaccharides (LPS). It acted as a neuroprotector to increase neuronal survival against microglia-induced neuron death [12]. Our results strongly supported rifampicin as a potential therapeutic for the treatment of neurodegenerative diseases. Despite the above findings, the mechanism through which rifampicin inhibits neuroinflammation is not completely understood.

NF-kB is an important transcription factor for the expression of pro-inflammatory mediators [13]. In unstimulated cells, nuclear factor-kappa B (NF-kB) binds to IkappaBalpha (IkBα) and its activity is inhibited. The activation of NF-kB is initiated by signal-induced degradation of IkBα proteins [14], which occurs primarily via the ubiquitin–proteasome pathway [15]. Proteasomes play a critical role in protein degradation and are essential to many intracellular processes [16]. The 26S proteasome, a multi-subunit enzyme complex, is a major cellular non-lysosomal protease. Modulation of the ubiquitin-26S proteasome system (UPS) with proteasome inhibitors has indicated possible efficacy for the treatment of neuroinflammatory disorders [17]. We used 2-dimensional gel electrophoresis (2-DE) and mass spectrometry (MS) to identify proteins affected by rifampicin in activated microglia. We uncovered that the expression of 26S protease regulatory subunit 7 (MSS1) was reduced. MSS1 localizes to both the nucleus and the cytoplasm. It functions as a chaperone-like subunit in the 19S regulatory complex and participates in intracellular proteasome events [18]. Based on the above evidence, we further hypothesized that rifampicin inhibited the expression of MSS1, thus suppressed IkBα degradation and the production of inflammatory mediators.

In this study, we examined the effect of rifampicin on the expression of MSS1 in LPS-stimulated BV2 microglia by western blot to confirm the results of proteomics experiments. We demonstrated that after silencing the expression of MSS1 gene via RNA interference (RNAi), IkBα protein degradation and NF-kB activity were both downregulated in LPS-stimulated BV2 microglia. We also showed that the production of inducible NO synthase (iNOS), NO, cyclooxygenase-2 (COX-2), and PGE_2_ were significantly decreased after MSS1 gene knockdown in LPS-activated BV2 microglia. Our results implied that rifampicin inhibited IkBα degradation by suppressing the expression of MSS1, therefore regulated the production of inflammatory mediators.

## Materials and Methods

### Chemicals and Reagents

Rifampicin (purity >98%), LPS and dimethyl sulfoxide (DMSO) were purchased from Sigma (St. Louis, MO). Rifampicin was dissolved in less than 0.1% of DMSO solution. Antibodies against iNOS, COX-2, and IkBα were obtained from Cell Signaling Tech (Beverly, MA). Mouse beta-actin antibody was purchased from Sigma. Dulbecco’s-modified Eagle’s medium (DMEM) containing L-arginine (200 mg/L), fetal bovine serum (FBS), and other tissue culture reagents were purchased from Gibco (Grand Island, NY).

### Cell Culture

BV2-immortalized murine microglial cells were provided by the Cell Culture Center of the Chinese Academy of Medical Sciences (China). Cells were cultured in DMEM supplemented with 10% FBS, 100 units/ml penicillin, and 100 µg/ml streptomycin in a humidified atmosphere of 5% CO_2_ at 37°C [12]. To examine the effect of rifampicin on the expression of MSS1 in LPS-stimulated BV2 microglia, 3×10^5^ cells per well were seeded in 6-well plates and pretreated with 150 µmol/L rifampicin for 2 hours (h) before the addition of LPS (1000 ng/ml).

### 2-dimensional Gel Electrophoresis and Image Analysis

LPS-treated cells were washed three times with ice-cold washing buffer (10 µM Tris-HCl, 250 µM sucrose, pH 7.0), collected in clean 1.5 ml eppendorf tubes. Lysis buffer [7 M urea, 2 M thiourea, 4% CHAPS (w/v), 1% dithiothreitol (DTT), 2% immobilized pH gradients (IPG) (v/v), pH 3–10 NL] was added, and samples were centrifuged at 13,200 *g* for 30 min at 4°C. The supernatant was subjected to 2-DE using an Amersham Biosciences IPGphor IEF System and Hoefer SE 600 (GE healthcare, Uppsala, Sweden) electrophoresis units (13 cm), according to manufacturer’s instructions and a previously described protocol [19]. Protein lysates and 2-DE gels were processed in parallel. Protein concentrations were determined using the Bradford assay. After 2-DE, the gels underwent silver nitrate staining according to a previously described protocol [20], then were scanned using an Image Scanner (GE Healthcare). The images were analyzed using the ImageMaster 2D Platinum (GE Healthcare).

### Matrix-assisted Laser Desorption/ionization Time-of-flight Mass Spectrometry (MALDI-TOF-MS) and Database Search

Only protein spots that were consistently different in at least three independent experiments were considered to be significant for analysis by MALDI-TOF-MS. Protein spots were excised from the silver-stained gels and transferred into siliconized 1.5 ml eppendorf tubes. Tryptic in-gel digestion was performed as previously reported with slight modifications [19]. Molecular mass analysis of the tryptic peptides was performed using ABI 4800 plus a MALDI-TOF-MS mass spectrometer (Applied Biosystems, Foster City, CA). Spectra were interpreted and processed using the Global Protein Server Workstation (V3.6, Applied Biosystems) via the internal MASCOT search engine (V2.1, Matrix Science, London, UK) to analyze MALDI-TOF-MS and MS/MS data. Based on combined MALDI-TOF-MS and MS/MS spectra, MASCOT protein scores of greater than 65 were considered statistically significant (*p*<0.05). The individual MS/MS spectrum with the best ion score (based on MS/MS spectra) that was statistically significant (*p*<0.05) was also accepted. Searches were performed against the IPI mouse database (V3.36) with parameters as the following: the enzyme trypsin with one missed cleavage was allowed; variable modifications included acetamidation of cysteine and oxidation of methionine; peptide mass tolerance was set to 50 ppm and fragment ion mass tolerance was set to 0.2 Da; and only monoisotopic masses were included in the search.

### MSS1 Gene silencing

Gene silencing was performed using small interference RNA (siRNA) targeting MSS1 mRNA for degradation. MSS1-specific siRNAs had the sense sequence of 5′-GUCGAACGCACAUCUUUAATT-3′, corresponding to a region that was 443–461 bases downstream of the first nucleotide of the start codon of mouse MSS1 cDNA (GenBank Accession Number: NC_000007.13). The sense sequence of scrambled siRNAs was 5′-UUCUCCGAACGUGUCACGUTT-3′. RNA duplexes were synthesized, purified and annealed by Dharmacon (Lafayette, CO). BV2 cells were transfected with targeting and scrambled RNA duplexes at a final concentration of 100 nM using Lipofectamine 2000 (Invitrogen, Grand Island, NY) in either 96-well or 6-well culture plates. The cells were assayed at 24 h post-transfection via western blotting.

### Nitrite (Griess) Assay

The NO levels in the culture supernatants were determined by measuring nitrite levels using a Griess reaction [21]. Six wells of cells were treated with rifampicin per experiment. After the BV2 microglial cells were stimulated in 24-well plates for 24 h, 100 µl of the cell culture medium was taken out and mixed with the same volume of the Griess reagent [1% sulfanilamide, 0.1% N-(1-naphthyl)-ethylenediamine dihydrochloride, 2.5% H_3_PO_4_]. The nitrite concentration was determined by evaluating the absorbance at 540 nm using a 96-well microplate spectrophotometer and calculated by referring to a standard curve.

### Enzyme-linked Immunosorbent Assay (ELISA)

The concentration of PGE_2_ in cell-conditioned culture medium was assessed using an ELISA kit (R&D Systems, Minneapolis, MN) according to the manufacturer’s instructions. Three wells of cells were treated per experiment.

### Western Blot Analysis

The BV2 microglial cells were harvested from each group and followed by western blot analysis that was conducted as previously described [22]. Cell pellets were briefly lysed in RIPA buffer [1 mM ethylenediaminetetraacetic acid (EDTA), 150 mM NaCl, 1% igepal, 0.1% sodium dodecyl sulfate (SDS), 0.5% sodium deoxycholate, and 50 mM Tris-HCl, pH 8.0]. Equal amounts of cellular proteins were separated by 8–12% sodium dodecyl sulfate polyacrylamide gel electrophoresis (SDS-PAGE), transferred to polyvinylidene fluoride (PVDF) membranes, blocked with 5% nonfat milk for 2 h, and incubated with antibodies against iNOS (1∶1000), COX-2 (1∶1000), IkBα (1∶1000), and β-actin (1∶5000) at 4°C overnight. The next day, the membrane was washed by Tris-Buffered Saline Tween-20 (TBST) three times, 10 minutes each, and incubated with the corresponding secondary antibodies that were horseradish peroxidase-conjugated for 1 h at room temperature. Antibody interactions were detected using enhanced chemiluminescence (ECL) followed by exposure to film.

### NF-kB Reporter Gene Assay

A total of 1×10^6^ BV2 microglial cells were transfected with 2 µg NF-kB-Luciferase reporter plasmid and pCMV-gal control vector (Clontech, Mountain View, CA) using Lipofectamine reagents according to the manufacturer’s protocol (Invitrogen). BV2 microglial cells were plated in 96-well plates at a density of 1×10^4^ cells per well and cultivated at 37°C overnight. Three wells of cells were treated per experiment. After incubation with the appropriate DNA-Lipofectamine mixtures, cells were pre-incubated with or without rifampicin for 2 h before the addition of LPS for 6 h. Cells were then washed, lysed, and centrifuged according to the manufacturer’s instructions (Promega, Madison, WI). 20 µl of cell extract was mixed with 100 µl of luciferase assay reagent at room temperature followed by the luciferase activity detection using a luminometer (Safire2, Tecan Instruments, Switzerland). Luciferase activity was normalized through dividing the mean luciferase relative light units (RLU) by the mean value of β-galactosidase RLU.

### Statistical Analysis

Data were presented as the mean ± standard error of the mean (SEM) derived from three or more independent experiments. Comparisons between two groups were analyzed using Student’s t-test. A value of *p*<0.05 was deemed to be statistically significant.

## Results

### 2-DE Maps and Protein Identification by MALDI-TOF-MS

**Figure 1:**
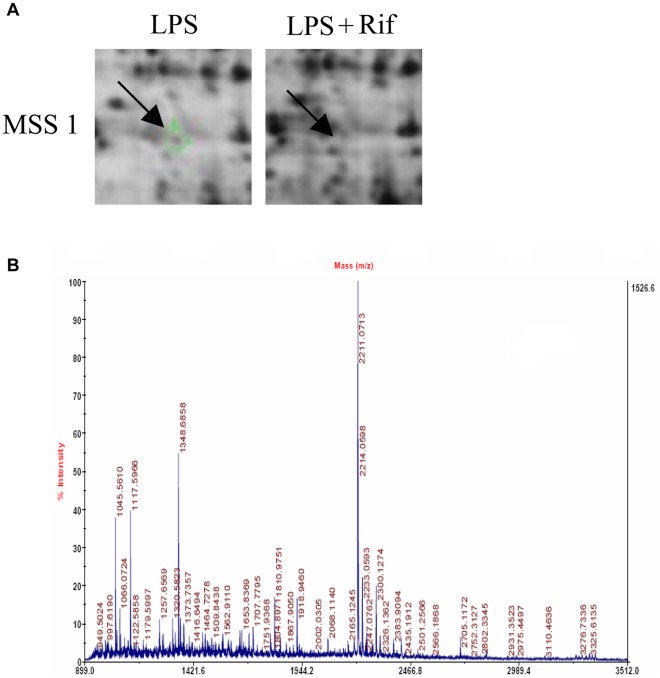
2D-DIGE gel images of proteins isolated from LPS-stimulated BV2 microglia with or without rifampicin pretreatment. Arrows indicate proteins that were differentially expressed in rifampicin-treated cells compared with non-treated controls. Peptide mass fingerprint spectra produced by MALDI-TOF-MS. Representative spectra from three independent experiments are shown. The x-axis represents mass-to-charge ratio (m/z), and the y-axis represents relative abundance. The peptide masses are labeled and annotated with corresponding m/z.

After matching, fifteen protein spots were extracted, digested, and submitted for identification by MALDI-TOF-MS. MSS1 protein was successfully identified. Its expression level was downregulated compared to the control (Figure 1). Detailed information about MSS1 proteins is listed in Table 1, including International Protein Index (IPI) accession number, molecular weight, pH indicated, and rifampicin treated-to-vehicle fluorescence ratios.

**Table 1:** Differential MSS1 protein expression identified by MALDI-TOF-MS.

Accession Number	Name	Molecular Weight (Dalton)	pH Indicated	Ratio of Spot Density(Rifampicin/Vehicle)
IPI 00270326	26S proteasomeregulatory subunit 7	52,867	5.97	−1,000,000

### Rifampicin Significantly Suppressed the Expression of MSS1 in LPS-activated BV2 Microglia

**Figure 2:**
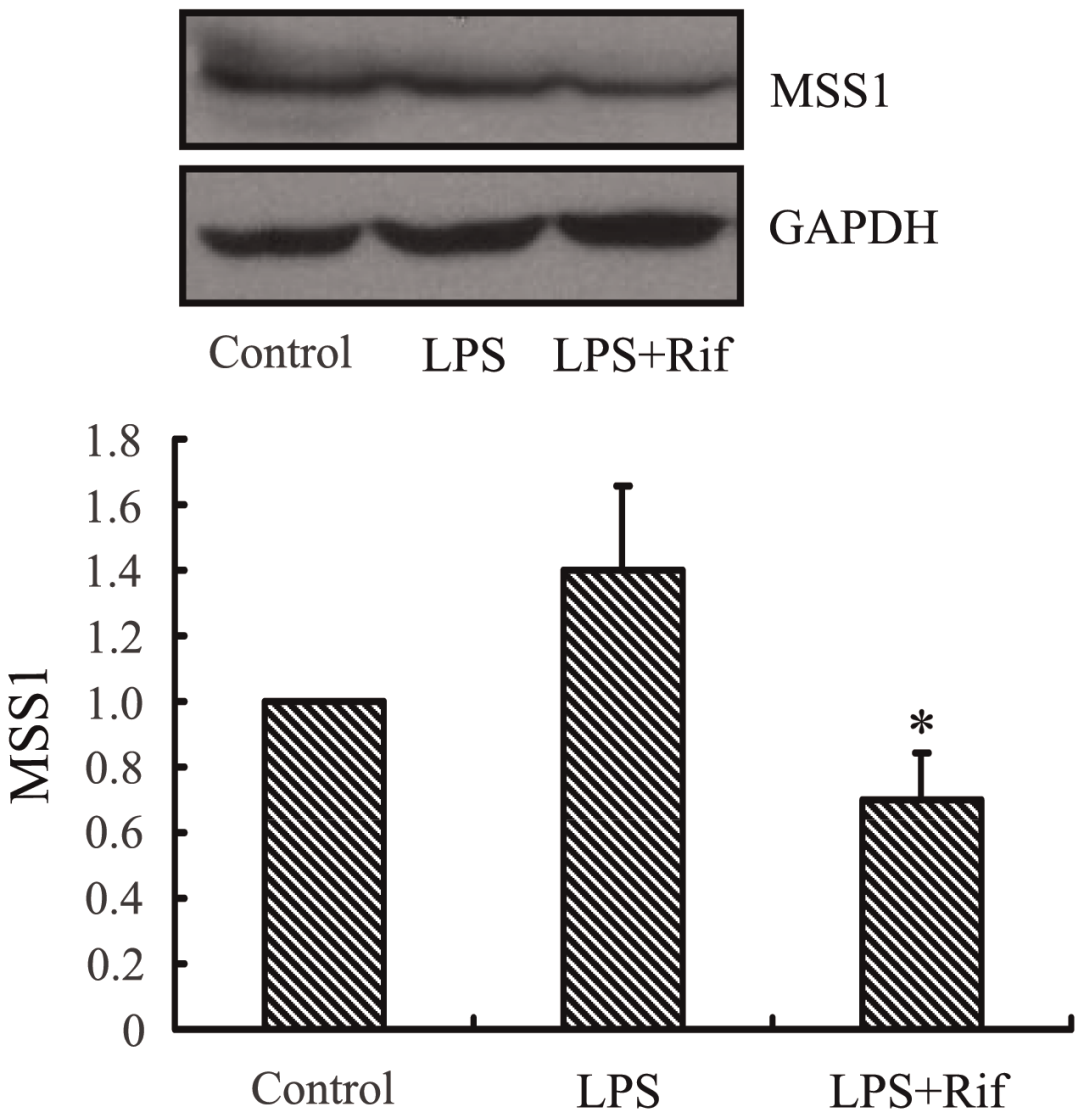
Rifampicin significantly suppressed the expression of MSS1 in LPS-stimulated BV2 microglia. Cells were treated with the indicated doses of rifampicin for 2 h prior to the addition of LPS (1000 ng/ml). At 24 h post-LPS incubation, cell lysates were analyzed for the protein production of MSS1 using western blot. Rifampicin significantly inhibited the LPS-induced MSS1 expression at protein levels. Data were obtained from three independent experiments with four to six replicates each. *p<0.05 compared with untreated cells and cells treated with LPS in the absence of rifampicin.

To examine the effect of rifampicin on the expression of MSS1 in LPS stimulated BV2 microglia, we measured the protein levels of MSS1 in LPS-stimulated BV2 microglia. Rifampicin treatment greatly inhibited the LPS-induced MSS1 protein expression (Figure 2). The result suggested that rifampicin significantly reduced the expression of MSS1 in LPS-stimulated BV2 microglia.

### Verification of MSS1 Gene Silencing

**Figure 3:**
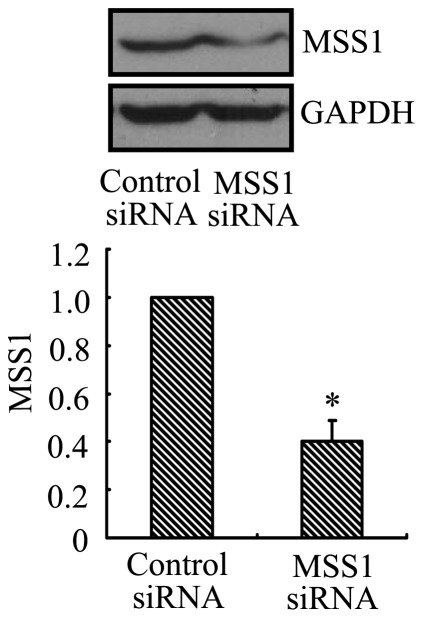
MSS1 gene knockdown reduced the expression of MSS1 at protein levels. In order to assess the efficacy of gene silencing, western blot analysis was performed after transfection with siRNAs targeting MSS1. The specificity of MSS1 gene silencing was determined by comparing with cells transfected with the scrambled RNA duplex. The BV2 cells were transfected with either MSS1-specfic or control siRNAs. At 24 h post-incubation, cell lysates were analyzed for the protein expression of MSS1 using western blot. Compared with the negative control group, the expression of MSS1 was significantly reduced by incubation with MSS1-targeted siRNAs. Data were obtained from three independent experiments with four to six replicates each. *p<0.05 compared with the negative control group.

We used western blot analysis to confirm the gene knockdown of MSS1 by its targeting siRNAs. After transfection with siRNAs, the expression of MSS1 protein was decreased to 40% compared with the negative control cells. The difference was statistically significant (*p*<0.01, Figure 3).

### IkBα Protein Degradation was Significantly Reduced by MSS1 Gene Knockdown in LPS-stimulated BV2 Microglia

**Figure 4:**
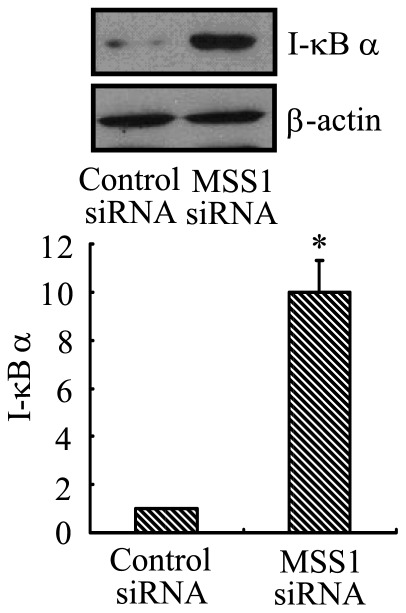
MSS1 gene silencing decreased IkBα protein degradation in LPS-activated microglia. The BV2 cells were transfected with either MSS1-specfic or control siRNAs for 24 h, then cells were stimulated with LPS (1000 ng/mL) for 30 min before cell lysates were analyzed for IkBα expression using western blot. IkBα protein degradation was significantly reduced after the addition of siRNAs targeting MSS1 in LPS-induced BV2 microglia. Data were obtained from three independent experiments with four to six replicates each. *p<0.05 compared with the negative control group.

To examine the regulation IkBα degradation by MSS1 in LPS-activated microglia, the BV2 cells were transfected with either MSS1-specfic or control siRNAs for 24 h followed by LPS stimulation at 1000 ng/mL for 30 min. Cell lysates were analyzed for the protein expression of IkBα using western blot. Our results demonstrated that IkBα protein degradation was significantly reduced after MSS1 gene knockdown in LPS-stimulated BV2 microglia (Figure 4).

### Downregulation of Microglial NF-kB Activation by MSS1 Gene Silencing in Response to LPS Stimulation

**Figure 5:**
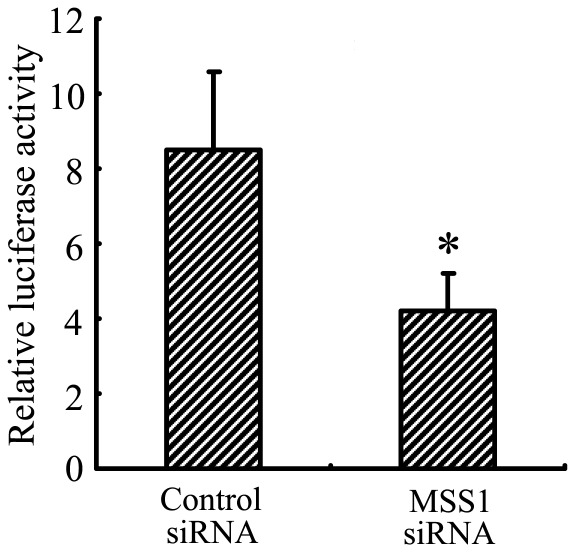
MSS1 gene silencing inhibited microglial NF-kB activation in response to LPS stimulation. The BV2 cells were transfected with either MSS1-specfic or control siRNA for 24 h, then cells were incubated with LPS at 1000 ng/mL for 8 h. After that, cells were transfected with NF-kB-luciferase reporter plasmid and pCMV-gal control vectors using Lipofectamine reagents. NF-kB activation was detected and expressed as relative luciferase activity. Compared with the negative control group, treatment with MSS1-targetd siRNA significantly suppressed the enhancement of NF-kB activity by LPS. Data were obtained from three independent experiments with four to six replicates each. *p<0.05 compared with the negative control group.

After MSS1 gene knockdown via RNAi, we assessed NF-kB activation using the NF-kB reporter gene assay. The BV2 cells were transfected with either MSS1-specfic or control siRNAs for 24 h. Cells were then incubated with LPS at 1000 ng/mL for 8 h. NF-kB activity was determined by measuring the relative luciferase activity. As shown in Figure 5, LPS markedly enhanced NF-kB activity, while transfection with MSS1-targeted siRNA significantly inhibited the enhancement.

### Decrease of iNOS Expression and NO Production by MSS1 Gene Knockdown in LPS-induced BV2 Microglia

**Figure 6:**
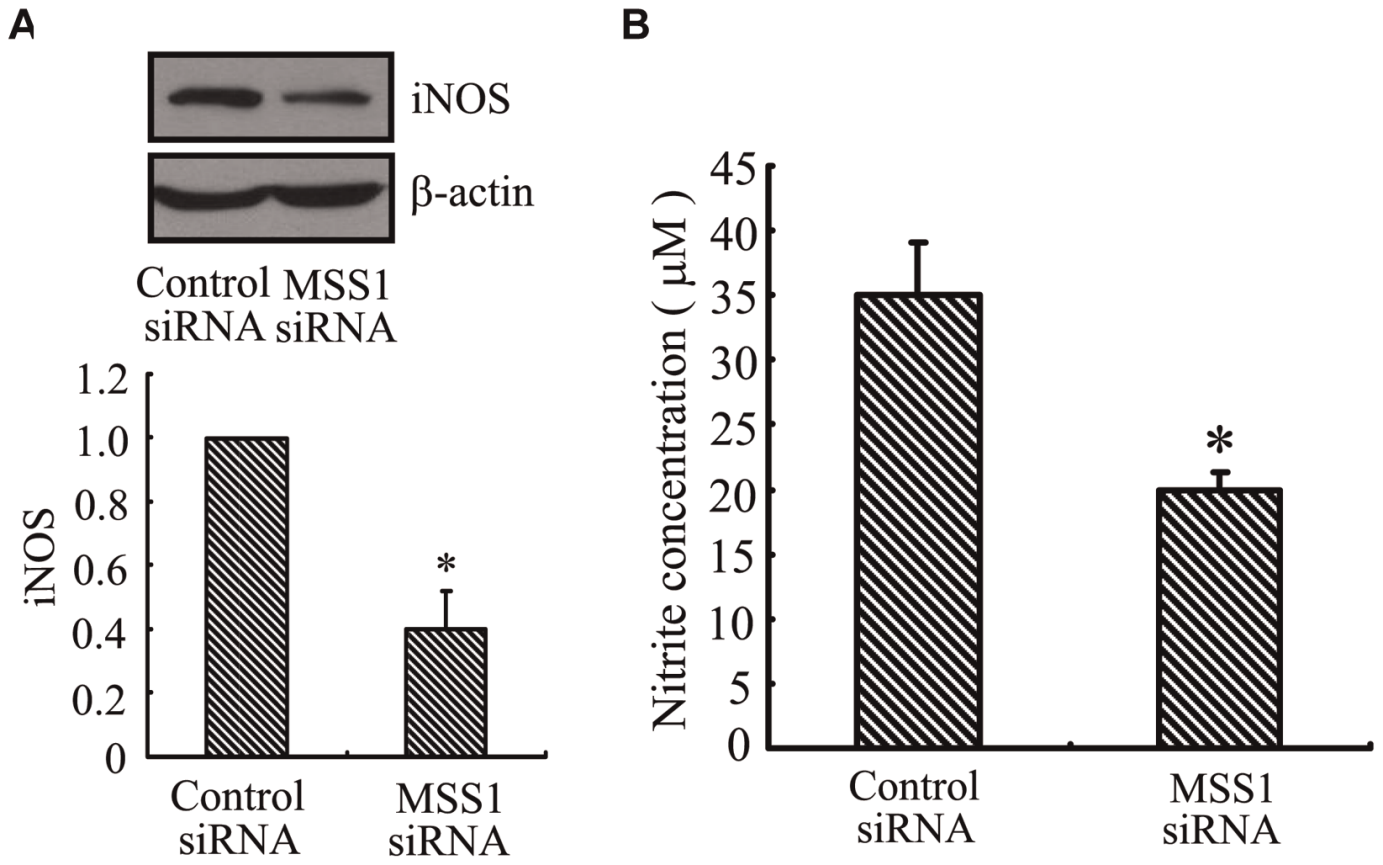
Decreased iNOS expression and NO production by MSS1 gene silencing in LPS-activated microglia. The BV2 cells were transfected with either MSS1-specfic or control siRNA for 24 h, then cells were stimulated for 24 h with LPS (1000 ng/mL). At 24 h post-LPS incubation, cell lysates were analyzed for the protein production of iNOS using western blot. The Griess assay was performed to measure the production of the NO metabolite, nitrite. Transfection with MSS1-specific siRNA suppressed the LPS-induced iNOS expression at protein levels, along with the production of nitrites Data were obtained from three independent experiments with four to six replicates each. *p<0.05 compared with the negative control group.

To investigate the effect of MSS1 gene silencing on iNOS expression and NO production, we measured their protein levels as well as the accumulation of nitrite, a stable metabolite of NO, in LPS-stimulated BV2 microglia. Transfection using MSS1-specific siRNA greatly inhibited the LPS-induced iNOS protein expression (Figure 6). We next evaluated the NO production in culture supernatants by detecting nitrite levels using a Griess reaction. Consistent with the downregulation of iNOS, transfection with MSS1-targeted siRNAs reduced the LPS-mediated NO production in BV2 microglia (Fig. 6). Our results indicated that MSS1 gene silencing suppressed the production of pro-inflammatory NO by inhibiting the expression of iNOS in LPS-stimulated BV2 microglia.

### Inhibition of COX-2 Expression and PGE_2_ Production by MSS1 Gene Knockdown in LPS-activated BV2 Microglia

**Figure 7:**
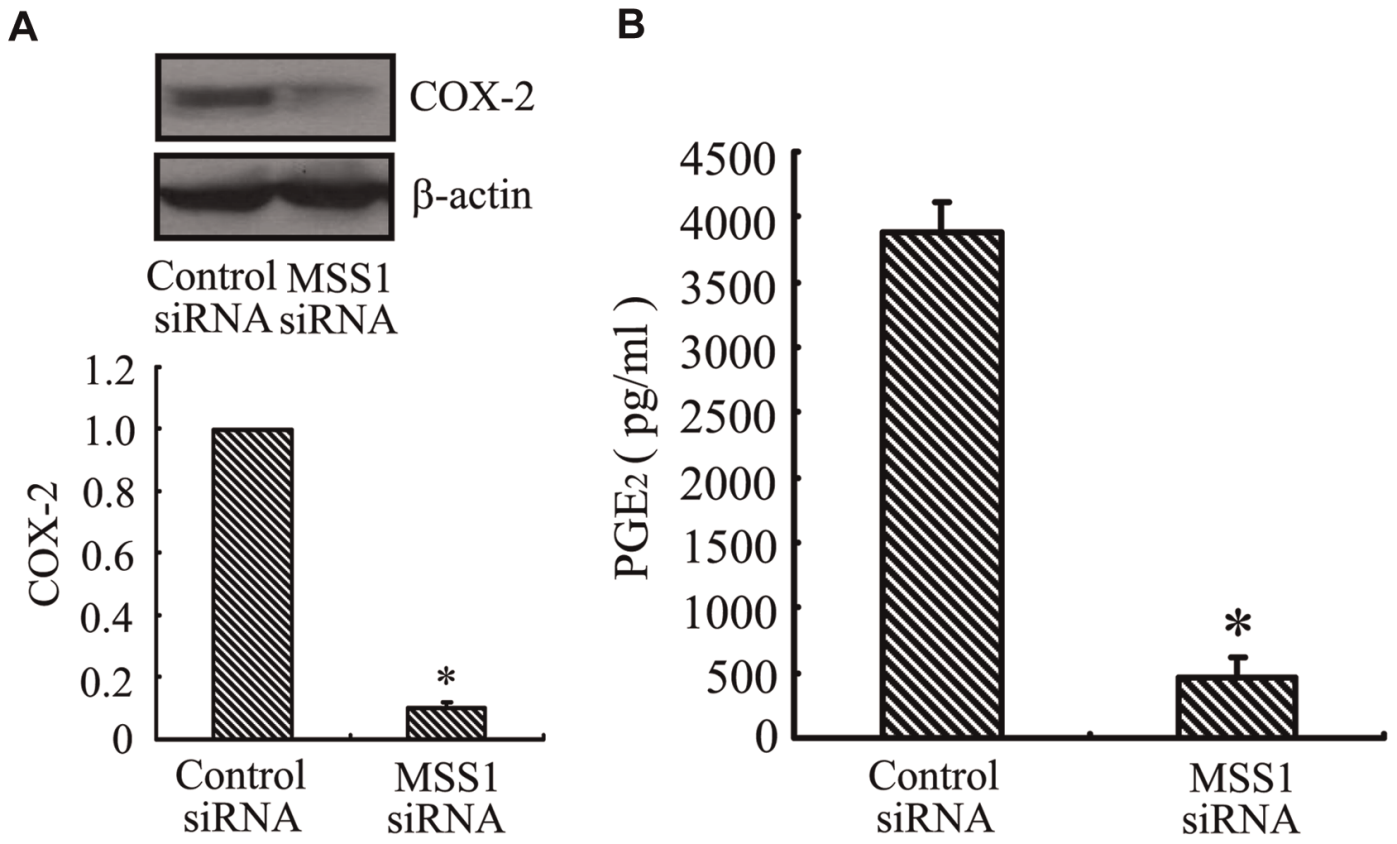
Inhibition of COX-2 expression and PGE_2_ production by MSS1 gene knockdown in LPS-induced microglia. The BV2 cells were transfected with either MSS1-specfic or control siRNA for 24 h, then cells were stimulated for 24 h with LPS (1000 ng/mL). At 24 h post-LPS incubation, cell lysates were analyzed for the protein production of COX-2 using western blot. We collected the supernatant and further analyzed the production of PGE_2_. MSS1 gene silencing suppressed the LPS-induced COX-2 expression at protein levels, as well as PGE_2_ production. Data were obtained from three independent experiments with four to six replicates each. *p<0.05 compared with the negative control group.

To address the effect of MSS1 gene silencing on COX-2 and PGE_2_ production, we assessed their expression in LPS-induced BV2 cells. Transfection using MSS1-targeted siRNAs significantly inhibited the LPS-induced COX-2 protein expression (Figure 7). We next collected the supernatant and analyzed the concentration of PGE_2_ using ELISA. Transfection with MSS1-specific siRNAs decreased the LPS-induced PGE_2_ production in BV2 microglia (Figure 7). These results implied that MSS1 gene silencing suppressed the production of pro-inflammatory PGE_2_ by inhibiting the expression of COX-2 in LPS-stimulated BV2 microglia.

## Discussion

The immunosuppressive properties of rifampicin have been discussed in the literature for 30 years [23]–[27]. Calleja *et al*. discovered that rifampicin activated the human glucocorticoid receptor (hGR), which regulated the expression of various genes including those that encoded interleukins [28]. Further investigations uncovered that rifampicin inhibited Toll-like receptor 2 (TLR2) via the suppression of the DNA binding of NF-kB, providing a novel mechanism contributing to the immunosuppression of rifampicin [29]. Our results showed that the anti-inflammatory, neuroprotective properties of rifampicin were mediated through the inhibition of signaling molecules, such as NF-kB and mitogen activated protein kinases (MAPKs) in LPS-activated BV2 microglial cells [12]. However, the mechanism by which rifampicin reduces microglial inflammation is not completely understood.

In this investigation, we used 2-DE and MALDI-TOF-MS to identify proteins affected by rifampicin in LPS-pretreated microglia. We successfully identified MSS1 protein and showed that its expression was downregulated (Figure 1A and 1B). The proteomic results were verified by western blot analysis, which also demonstrated the inhibition of the LPS-induced MSS1 expression by rifampicin (Figure 2). Our findings suggested that MSS1 was involved in microglial inflammation and its gene knockdown by RNAi alleviated neuroinflammation (Figure 3) in LPS-activated BV2 cells. Our results open the door to further study MSS1 as a potential therapeutic target for neuroinflammation.

UPS represent an ATP-dependent protein degradation mechanism in eukaryotic cells. The 26S proteasome is a multi-catalytic proteinase complex. It has a highly ordered structure composed of two complexes, a 20S core and a 19S regulator. The 19S regulator is composed of a base, which contains 6 ATPase subunits and 2 non-ATPase subunits, and a lid, which contains up to 10 non-ATPase subunits [30]. Proteasomes are distributed throughout eukaryotic cells at a high concentration and cleave peptides in an ATP/ubiquitin-dependent process in a non-lysosomal pathway [31]. Alterations in UPS are correlated with a variety of human pathologies, such as cancer, immunological disorders, inflammation, and neurodegeneration [31]–[33].

MSS1, also known as S7 or PSMC2, is a 433-amino-acid member of the AAA ATPase family. As a chaperone-like subunit of the 19S regulatory complex, MSS1 localizes in both the nucleus and the cytoplasm where it participates in proteasome events throughout the cell [18]. Additionally, MSS1 is thought to interact with several basal transcription factors and, via this interaction, play a role in transcriptional regulation [34]. Our data provided strong evidence for a novel mechanism of MSS1-mediated neuroinflammation. Additional investigations are needed to further elucidate this pathway and address the potential of MSS1 gene silencing as a therapy for neurodegenerative disorders.

NF-kB is an important transcription factor for the secretion of pro-inflammatory mediators [35]. LPS is shown to increase NF-kB activation, through IkB phosphorylation and the subsequent IkB degradation in macrophages [36]. In the canonical pathway of NF-kB induction, IkBs are phosphorylated at two amino-terminal serines, thus targeting them for polyubiquitination and the subsequent proteasomal degradation. IkB degradation enables NF-kB to translocate to the nucleus and bind to its target genes, including IkB. In addition, proteasomal degradation of transcriptionally active p65/RelA promotes the prompt termination of NF-kB responses [37]. The ubiquitin–proteasome pathway is considered pivotal to signal-induced IkB degradation [38]–[39]. In this study, we investigated the regulation of IkB and NF-kB signaling pathways by MSS1 gene silencing using a reporter gene assay and western blot analysis. Our results demonstrated that LPS caused rapid degradation of IkBα, while MSS1 gene knockdown significantly reduced IkBα degradation in LPS-stimulated BV2 microglia (Figure 4). We also found that LPS markedly enhanced NF-kB activity, whereas treatment with MSS1-specific siRNA significantly inhibited the enhancement (Figure 5). These results suggested that MSS1 gene knockdown suppressed NF-kB activation, likely through the blockage of IkBα degradation. The downregulation of IkBα provided a novel mechanism for MSS1’s immunomodulation in LPS-activated microglia.

Intranuclear blockage of NF-kB has been reported to suppress the expression of iNOS and COX-2 [40]. Our results indicated that transfection with MSS1-targeted siRNAs decreased the production of pro-inflammatory NO by inhibiting the expression of iNOS in LPS-stimulated BV2 microglia (Figure 6A and 6B). We also found that gene knockdown of MSS1 reduced the production of pro-inflammatory PGE_2_ through suppressing COX-2 gene expression in LPS-induced BV2 microglia (Figure 7A and 7B).

In conclusion, we demonstrated that rifampicin inhibited the expression of MSS1, which subsequently decreased IkBα degradation and the production of inflammatory mediators. Our results supported the potential application of MSS1 suppression, together with anti-inflammatory rifampicin, for the treatment of neuroinflammation and neurodegeneration.
